# A feasibility Randomised Controlled Trial as a first step towards evaluating the effectiveness of a digital health dashboard in preventive child health care: a mixed methods approach

**DOI:** 10.1186/s40814-022-01214-w

**Published:** 2023-02-15

**Authors:** Miriam Weijers, Nicolle Boumans, Jonne van der Zwet, Frans Feron, Caroline Bastiaenen

**Affiliations:** 1 Department of Social Medicine, Faculty of Health, Medicine and Life Sciences, Care and Public Health Research Institute, Maastricht University, P.O. Box 616, 6200 MD Maastricht, the Netherlands; 2 Department of Epidemiology, Faculty of Health, Medicine and Life Sciences, Care and Public Health Research Institute, Maastricht University, Maastricht, the Netherlands

**Keywords:** Complex intervention, Evaluation, RCT feasibility studies, Child health services, Prevention, International classification of functioning, Disability and health

## Abstract

**Background:**

Within preventive Child Health Care (CHC), the 360°CHILD-profile has been developed. This digital tool visualises and theoretically orders holistic health data in line with the International Classification of Functioning, Disability and Health. It is anticipated that evaluating the effectiveness of the multifunctional 360°CHILD-profile within the preventive CHC-context is complex. Therefore, this study aimed at investigating the feasibility of RCT procedures and the applicability of potential outcome measures for assessing the accessibility and transfer of health information.

**Methods:**

During the first introduction of the 360°CHILD-profile in CHC practice, a feasibility RCT with an explanatory-sequential mixed methods design was executed. CHC professionals (*n*=38) recruited parents (*n*=30) who visited the CHC for their child (age 0–16). Parents were randomised to “care as usual” (*n*=15) or “care as usual with, in addition, the availability of a personalised 360°CHILD-profile during 6 months” (*n*=15). Quantitative data on RCT feasibility were collected on recruitment, retention, response, compliance rates and outcome data on accessibility and transfer of health information (*n*=26).

Subsequently, thirteen semi-structured interviews (5 parents, 8 CHC professionals) and a member check focus group (6 CHC professionals) were performed to further explore and gain a deeper understanding of quantitative findings.

**Results:**

Integration of qualitative and quantitative data revealed that the recruitment of parents by CHC professionals was problematic and influenced by organisational factors. The used randomisation strategy, interventions and measurements were executable within the setting of this specific study. The outcome measures showed skewed outcome data in both groups and a low applicability to measure accessibility and transfer of health information. The study revealed points to reconsider regarding the randomisation and recruitment strategy and measures in the next steps.

**Conclusions:**

This mixed methods feasibility study enabled us to gain a broad insight into the feasibility of executing an RCT within the CHC context. Trained research staff should recruit parents instead of CHC professionals. Measures, potentially for evaluating 360°CHILD-profile’s effectiveness, need further exploration and thorough piloting before proceeding with the evaluation process. Overall findings revealed that executing an RCT within the context of evaluating 360°CHILD-profile’s effectiveness in the CHC setting will be much more complex, time-consuming and costly than expected. Thereby, the CHC context requires a more complex randomisation strategy than executed during this feasibility study. Alternative designs including mixed methods research must be considered for the next phases of the downstream validation process.

**Trial registration:**

NTR6909; https://trialsearch.who.int/.

**Supplementary Information:**

The online version contains supplementary material available at 10.1186/s40814-022-01214-w.

## Key messages regarding feasibility

### Uncertainties regarding feasibility that existed prior to this study

It was not known whether RCT procedures would be feasible within the organisational context. Furthermore, it was not known whether the chosen outcome measures potentially were applicable to an RCT aiming at the pre-defined outcome information.

### Key feasibility findings from this study

This study revealed that, within the setting of the CHC, it will be very complex, time-consuming and costly to use an RCT with the aim to compare the chosen intervention as add-on with care as usual. Several organisational factors within the CHC played a role; the organisational structure with one physician being responsible for a certain group (area of parents) hindered the most optimal randomisation strategy; insufficient facilitation and support from the organisation for this research project hindered recruitment of parents by CHC professionals. Applicability of the used measures was limited as the used answer options were very skewed in one direction and relevant items on accessibility and transfer of health information appeared to be applicable for a small number of participants.

### Implications of the findings for the design of the main study

For future studies on the 360°CHILD-profile’s effectiveness, participating professionals should be sufficiently facilitated and familiarised with the 360°CHILD-profile. Professionals should be foremost capable to perform the intervention, while recruitment of parents should be performed by trained research staff. Researchers should explore and pilot more potential measures for generating outcomes, applicable for assessing 360°CHILD-profile’s effectiveness within CHC. To prevent the postponement of the implementation of the promising 360°CHILD-profile in CHC practice, alternative designs and mixed methods research must be considered with a focus on generating outcomes that are needed for deciding if and how to proceed with the implementation and evaluation process.

## Background

Within the practice of the Dutch preventive Child Health Care (CHC), children’s health and development are systematically monitored. CHC professionals focus on protecting and promoting children’s health. For preventive clinical reasoning, it is essential to gain an integral overview of the collected health information and theoretically structure health data. Access to relevant health data, registered within the Electronic Medical Dossier (EMD), is currently hindered due to its non-theoretical structure and lack of overview. Therefore, together with fellow CHC medical doctors and researchers, the first author initiated the development of a digital health dashboard, the first version of the 360°CHILD-profile [1]. The actual dashboard visualises and theoretically orders holistic health data based on the International Classification of Functioning, Disability and Health, Children and Youth version (ICF-CY) [2, 3]. The 360°CHILD-profile is designed to facilitate the CHC’s transfer of health information to parents and youth, clinical reasoning processes, tailored counselling and shared decision-making. Although this digital dashboard is promising to offer the CHC a multifunctional tool [1], it is not yet known how it meets expectations in real-life practice.

During an iterative mixed methods design process, qualitative development processes were followed up with a quantitative validation process and again sequential qualitative processes to improve the design until the final representation was reached to be used in the actual feasibility study. International standards for representing health information were applied during the design of the 360°CHILD-profile. During the whole trajectory, professionals of different backgrounds and parents were actively involved and evaluation methods were integrated during the development of the 360°CHILD-profile to achieve a solid and applicable visualization with a high probability to perform as intended [1, 4, 5]. As described in detail elsewhere [1], a nested design model adapted from Munzner was used for guidance on how to integrate design and evaluation methods within each level of the design process. This model provides insight into how and when to apply quantitative- and qualitative evaluation methods during the developmental phase (i.e. upstream validation), as well as during the implementation of the delivered data-visualization design (i.e. impact-oriented downstream validation) [4].

So far, the 360°CHILD-profile’s downstream validation process included pilot tests of the delivered prototype. These showed positive results on comprehensibility, acceptability, reliability and validity [1, 6].

The next steps within the downstream validation process were to evaluate the implementation, usability and effectiveness of the 360°CHILD-profile within real-life CHC practice [1, 4]. Evaluating the effectiveness of the 360°CHILD-profile in the preventive CHC context was expected to be complex because the tool has multiple functions. Thereby, the effects of preventive interventions (especially effects at the level of health outcomes) cannot all be expected to be evident shortly after implementing an innovative tool. Moreover, the target group was heterogeneous as it included parents of children from 0 to 18 years old, with a normal development until a development characterized by severe health problems; parents with different educational levels, birth country or experienced levels of parental stress; and health care professionals including different disciplines and professional experience. To prepare for a solid evaluation of the effectiveness of this promising tool, it was essential to timely address feasibility questions regarding how to set up robust effect studies [7–9]. Before spending much time and effort in executing an RCT, it is important to ensure the availability of appropriate recruitment strategies, randomisation plans and outcome measures that are suitable for capturing significant differences of interest between the experimental- and control interventions [9, 10]. A feasibility RCT, integrating quantitative- and qualitative methods, enables a thorough evaluation of the practicability and acceptability of methodological conditions of an RCT within the given context [8–10]. Moreover, valuable knowledge can be generated on organizational factors that potentially interfere with the performance of a methodological well-designed RCT.

To initiate the evaluation of the prerequisites of studying 360°CHILD-profile’s effectiveness within real-life healthcare practice, a feasibility RCT was executed [9]. This study, which evaluated methodological aspects of setting up an RCT, was part of a larger mixed methods research project that also studied the implementation and usability of the 360°CHILD-profile, which is published elsewhere [11].

The feasibility RCT was performed during an early stage of 360°CHILD-profile’s implementation. Therefore, the focus was on the most immediate expected outcomes of implementing this experimental intervention within the CHC: better access to electronic health data and a more comprehensible transfer of health information to parents [1, 12, 13].

Considering the actual phase of the downstream validation process, the following objectives were formulated:

- 1. To evaluate the feasibility of RCT procedures within the given organisational context of the Dutch CHC (recruitment, retention, response, compliance to the allocated intervention and measure completion) and define (if possible) points for improvements regarding the procedures.
- 2. To explore measures as potential conditional outcomes related to accessibility and transfer of CHC’s health information:
- Usability of the selected outcome measures within the CHC context
- The variance in the received outcome options of the measurement scale(s), and also in relation to a future sample size calculation within the target population.

## Methods

### Study design

This research with an explanatory-sequential mixed methods design included a quantitative and subsequential qualitative part [11, 14, 15]. Within a small-scale RCT, quantitative measurements were performed. Then, qualitative methods (semi-structured interviews and focus groups) were used to gain a deeper understanding of the quantitative findings regarding the feasibility of performing an RCT [11, 14, 15]. Our reporting follows the CONSORT guidelines (see CONSORT checklist in supplemental files) [16].

Integration of quantitative and qualitative data took place at different levels. Quantitative data were used to direct the sampling strategy of the qualitative part and for refining topic lists. Furthermore, during qualitative analysis, the quantitative findings were considered and intensively compared in the discussion of overarching themes/categories [11].

### Study population

All nurses and medical doctors from the local CHC departments in the Southern part of the Netherlands were eligible and invited to participate in the study with the aim to recruit at least 30 CHC professionals [17]. No further exclusion criteria were applied. After an information and instruction meeting and an informed consent procedure, the group of participating CHC professionals became responsible for recruiting parents. It was aimed to include at least 30 parents who visited the CHC with their child between the age of 0 and 16 years. Additionally, other caregivers involved in the care of these children and the children (adolescents) aged between 11 and 16 also could be invited to participate. There were no exclusion criteria, besides the presence of barriers that could hinder the profile’s readability (like a substantial language barrier or severe cognitive disorder).

For the qualitative part of this study, subgroups of participating CHC professionals and parents were selected. Purposive sampling was used to obtain heterogeneous subgroups [18] with contrasting characteristics (CHC professionals: discipline, experience within the CHC, satisfaction about the electronic medical dossier (EMD); parents: parental stress, educational level, birth country, opinion on CHC, and their child’s age and level of functioning).

### Randomisation and concealed allocation

Within the actual organisational structure of the Dutch CHC, choosing a proper method for randomisation turned out to be a challenge. The preferred randomisation program where each CHC professional only would be confronted with either the experimental or the control intervention was not possible without using a cluster randomisation schedule. As such a schedule would require multiplying the sample size of participants, this option was not possible within this feasibility study. Randomisation was performed at the level of individual parents by an independent person, who used a central block randomisation application to assign recruited parents to one of the two study arms (with blocks of 4 and 6) [11].

### Interventions

During the intervention period of 6 months, all parents received care as usual. A personalised 360°CHILD-profile was generated for the experimental group, directly after completing the informed consent and randomisation procedure. During the subsequent consultation, CHC professionals discussed this 360°CHILD-profile with parents. Within the intervention period, the personalised 360°CHILD-profiles were accessible for CHC professionals within the Electronic Medical Dossier (EMD) and for parents via an online portal. Both CHC professionals and parents were free to consult the profile anytime and/or use it for other tasks and/or in contact with other involved caregivers. Parents of the control group received a personalised 360°CHILD-profile 6 months after baseline, after completing the intervention period including all RCT outcome measurements.

### Measurements

#### Baseline characteristics of participants

At baseline, demographics and other characteristics were collected from CHC professionals, parents and their children (Tables 1 and 2). The information on CHC professionals included discipline, education, their experience with the EMD and the 360°CHILD-profile, and their use of technologies to share health information with parents (like e-mail, WhatsApp). The information on parents included gender, educational level, birth country, their concerns about their child’s health/development, and parental stress [19]. The information on the children whose parents visited the CHC included age, gender, level of functioning [20, 21], and experienced health problems. Baseline measurements are described in more detail in the protocol article [11].

**Table 1:** Baseline characteristics of participating CHC-professionals

**Characteristics CHC-professionals**		**CHC-professionals**	
		Total group (*n*=38) Number (%)^*^	Subgroup (*n*=18): (included ≥ 1 parent) Number (%)^**^
**Discipline:**			
Nurse		20 (53)	6 (33)
Medical Doctor		18 (47)	12 (67)
**Age of target group they work with:**			
Children age 0-4y.		18 (47)	10 (56)
Children age 4-18y.		20 (53)	8 (44)
**Specific CHC-education:**			
No specific CHC-education		18 (47)	6 (33)
Introduction course CHC		4 (11)	3 (17)
Specialist CHC		16 (42)	9 (50)
**Experience within CHC:**			
<2 years		5 (13)	2 (11)
2-5 years		5 (13)	2 (11)
5-10 years		0	0
10-15 years		4 (11)	4 (22)
>15 years		24 (63)	10 (56)
**Providing parents with information in current care:**			
via computer:	(almost) always	30 (79)	14 (78)
	rather often	6 (16)	2 (11)
	sometimes	2 ( 5)	2 (11)
	(almost) never	0	0
via tablet:	(almost) always	5 (13)	2 (11)
	rather often	3 ( 8)	2 (11)
	sometimes	2 ( 5)	0
	(almost) never	28 (74)	14 (78)
via e-mail:	(almost) always	6 (16)	4 (22)
	rather often	14 (37)	8 (45)
	sometimes	11 (29)	2 (11)
	(almost) never	7 (18)	4 (22)
via WhatsApp:	(almost) always	0	0
	rather often	2 ( 5)	0
	sometimes	8 (21)	3 (17)
	(almost) never	28 (74)	15 (83)
**Use of technology during current consultations with parents:**			
via computer:	(almost) always	27 (71)	15 (83)
	rather often	3 ( 8)	1 ( 6)
	sometimes	5 (13)	2 (11)
	(almost) never	3 ( 8)	0
via tablet:	(almost) always	4 (10)	2 (11)
	rather often	1 ( 3)	0
	sometimes	4 (11)	1 ( 6)
	(most) never	29 (76)	15 (83)
via e-mail:	(almost) always	4 (10)	3 (17)
	rather often	14 (37)	6 (33)
	sometimes	12 (32)	5 (28)
	(almost) never	8 (21)	4 (22)
via WhatsApp:	(almost) always	0	0
	rather often	0	0
	sometimes	8 (21)	3 (17)
	(almost) never	30 (79)	15 (83)
**Opinion on current EMD**			
satisfied		3 ( 8)	0
rather satisfied		25 (66)	13 (72)
rather unsatisfied		9 (24)	5 (28)
unsatisfied		1 ( 2)	0
**Known with 360°CHILD-profile**			
very known		4 (11)	2 (11)
rather known		26 (68)	13 (72)
little known		7 (18)	2 (11)
not known		1 ( 3)	1 ( 6)
**Level of acquired experience with 360°CHILD-profile**			
high		0	0
rather high		3 ( 8)	1 ( 6)
low		11 (29)	9 (50)
no experience		24 (63)	8 (44)
**Opinion about possibility to use 360°CHILD-profile**			
positive		23 (61)	12 (67)
rather positive		14 (37)	6 (33)
rather negative		1 ( 2)	0
negative		0	0
**Opinion about possibility to use E-health**			
positive		16 (42)	9 (50)
rather positive		21 (55)	8 (44)
rather negative		1 ( 3)	1 ( 6)
negative		0	0
**Number of parents recruited/included**:		Recruited	Included
none		14 (37)	0
one		13 (34)	11 (61)
two-three		8 (21)	5 (28)
four-five		1 ( 2)	2 (11)
> five		2 ( 5)	0

^*^Completed baseline measures

^**^Completed baseline measures and included parents

**Table 2:** Baseline characteristics of participating parents

**Parents’ Characteristics**	**Total group** (*n*=28) Number (%)	**Control group: Usual Care** (*n*=13) Number (%)	**Intervention group: Usual care and 360°CHILDoc** (*n*=15) Number (%)
**Relation to child:**			
Mother	27 (96)	13 (100)	14 (93)
Father	1 ( 4)		1 ( 7)
**Age:**			
18-25 years	1 ( 4)	0	1 ( 6)
25-35 years	14 (50)	7 (54)	7 (47)
35-45 years	13 (46)	6 (46)	7 (47)
**Number of children:**			
1 child	9 (32)	3 (23)	6 (40)
2 children	18 (64)	9 (69)	9 (60)
3-4 children	0	0	0
5 or more children	1 ( 4)	1 ( 8)	0
**Education**^a^**:**			
Low	5 (18)	2 (15)	3 (20)
Medium	12 (43)	4 (31)	8 (53)
High	11 (39)	7 (54)	4 (27)
**Birth country:**			
Of participating parent: the Netherlands	26 (93)	13 (100)	13 (87)
other than the Netherlands	2 ( 7)	0	2 (13)
Of other parent: the Netherlands	24 (86)	11 (85)	13 (87)
other than the Netherlands	4 (14)	2 (15)	2 (13)
**Perceived physical health**			
Good	24 (86)	10 (77)	14 (93)
Mediocre	3 (11)	2 (15)	1 ( 7)
Poor	1 ( 3)	1 ( 8)	0
**Perceived mental health**			
Good	27 (96)	13 (100)	14 (93)
Mediocre	1 ( 4)	0	1 ( 7)
Poor	0	0	0
**NOSIK**^b^**(**parental stress)			
Below average	9 (53)	4 (50)	5 (56)
Average	5 (29)	3 (38)	2 (22)
Above average	2 (11)	1 (12)	1 (11)
High	1 ( 6)	0	1 (11)
* Missing values: (only applicable for age 2-13)*	11	5	6
**Rating of CHC** (on continuous scale of 0-10)			
(mean, SD)	8.1 (1.0)	8.0 (1.1)	8.1 (0.9)
**Children’s characteristics**			
**Gender**			
Boy	15 (54)	7 (54)	8 (53)
Girl	13 (46)	6 (46)	7 (47)
**Age**			
(mean in years, SD) (range of age)	3.9 (3.6) (0.3-2.3)	3.3 (2.7) (0.3-7.6)	4.5 (4.3) (0.3-12.3)
**CGAS-score**^c^			
Functioning (mean, SD)	71.8 (16.7)	72.4 (18.5)	71.2 (15.6)
**STEP**^d^			
Functioning (mean, SD)	12.1 ( 5.7)	11.0 (6.1)	13.1 (5.3)
Quality environment (mean, SD)	8.6 (4.3)	8.5 (4.7)	8.7 (4.0)
Level of needed care (mean, SD)	7.0 (3.9)	6.3 (4.2)	7.6 (3.7)
**Problems** (more domains possible)			
* Total children with one or more problem(s)*	*19 (86)*	*8 (62)*	*11 (73)*
- Psychosocial	6 (21)	3 (23)	3 (20)
- Physical	8 (29)	4 (31)	4 (27)
- Cognitive	6 (21)	2 (15)	4 (27)
- Family/upbringing	5 (18)	1 ( 8)	4 (27)
- Youth & Environment	6 (21)	2 (15)	4 (27)
**CHC-contacts** last 6 months			
1 time	11 (39)	7 (54)	4 (27)
2-3 times	11 (39)	4 (31)	7 (46)
> 3 times	6 (22)	2 (15)	4 (27)
Other caregivers involved	13 (46)	6 (46)	7 (47)
Characteristics CHC*-prof. involved with specific child/parent			
**Discipline**			
Nurse	10 (36)	5 (39)	5 (33)
Medical Doctor	18 (64)	8 (61)	10 (67)
Target group CHC*			
children age 0-4 year	19 (32)	9 (69)	10 (67)
children age 4-18 year	9 (68)	4 (31)	5 (33)

^*^CHC: preventive Child Health Care

^a^Low education: no education up to and including low vocational training.

Medium education: basic vocational training through middle management/specialist training

Higher education: upper secondary education up to and including doctoral degree at research universities

^b^NOSIK: Dutch short version of parenting Stress Index; parents’ perspective on an ordinal scale [19]

^c^CGAS: Children's Global Assessment Scale; professional's rating of child functioning: continuous scale

(from 0 to 100) [20]

^d^STEP: Dutch standardised professional's rating on a reversed continuous scale of Functioning (from 30 to 6),

Quality environment (from 25 to 5) and level of needed care (from 5 to 3) [21]

#### RCT procedures measures

During the execution of the RCT, the following variables were collected: number of invited and included participants, follow-up, reasons for dropout, measurement completion, missing data and compliance with allocation. The first author documented occurring problems and adaptations made to the procedures to address these problems.

#### Quantitative outcome measures

Outcomes regarding accessibility and transfer of health information were measured 6 months after baseline. The search for an appropriate outcome measure, validated within the Dutch CHC setting, led to the “Consumer Quality Index (CQI) for the preventive CHC” (based on the “Consumer Assessment of Healthcare Providers and Systems” (CAHPS®)) [22, 23]. To the CQI, fifteen applicable items from “Supplemental items on Health Information Technology” (HIT) (available via the CAHPS®-website) were added [23, 24]. Additionally, six original questions were developed and incorporated into the measurement procedures about relevant dimensions of the construct “access to healthcare and health information” (availability, accommodation, accessibility and acceptability) [25]. Those questions had answer options on a two to five-point scale [22–24]. Outcome measures are described in more detail in the protocol article [11].

#### Qualitative measures

Semi-structured interviews with CHC professionals and parents were conducted to explore new perspectives on the feasibility of the RCT procedures.

To obtain relevant information from the CHC professionals and parents, topic lists included questions about their view on the CHC, transfer of health information, and their experiences regarding their study participation. Topic lists were slightly customised for each individual participant, considering already available individual quantitative data.

During a “member check” focus group meeting, the most relevant findings and preliminary interpretations were presented to professionals that joined the first rounds of interviews. CHC professionals were asked whether the findings and interpretations reflected their experiences and to further elaborate on and/or explain those findings [26, 27].

Both, the interviews and the subsequent focus group meeting were audio recorded (after explicit informed consent) and transcribed verbatim.

### Analysis

#### Baseline characteristics of participants

For baseline characteristics of CHC professionals and parents, descriptive analyses were performed. Characteristics of CHC professionals are presented for the total group that initially volunteered to participate as well as for the subgroup of professionals that included parents and actively participated during all phases within the RCT (Table 1). Characteristics of parents are presented for the total group and each randomised group separately (Table 2). Participant characteristics of the selected subsamples for the qualitative part are presented in a separate table (Table 3).

**Table 3:** Parents and CHC-professionals, participating in the semi-structured interviews

**Parents**	**Child’s age group**	**Child functioning** (STEP ^a^) 6-30 (high-low)	**Parental stress** (NOSIK ^b^)	**Educational level**	**Birth country**	**Rating of CHC** 0-10
1	4-18	11	< average	medium	other than the Netherlands	8
2	0-4	21	< average	high	the Netherlands	10
3	0-4	19	> average	low	the Netherlands	8
4	0-4	6	average	high	the Netherlands	8
5	4-18	-	-	medium	the Netherlands	-
**CHC-profes-sionals**	**Target age group**	**Discipline**	**Experience in CHC**	**Satisfaction about EMD**		
1	0-4	medical doctor	>15y	rather satisfied		
2 ^*^	4-18	nurse	0-5y	rather unsatisfied		
3 ^*^	0-4	nurse	>15y	rather satisfied		
4 ^*^	4-18	medical doctor	>15	satisfied		
5 ^*^	4-18	medical doctor	0-5 y	rather satisfied		
6 ^*^	0-4	medical doctor	>15y	satisfied		
7 ^*^	0-4	nurse	5-10y	-		
8	4-18	medical doctor	>15 y	satisfied		

^a^ STEP functioning score: Dutch standardised professional's rating of child's functioning on a (reversed) continuous scale[21]

^b^ NOSIK: Dutch short version of parenting Stress Index; parents’ perspective on an ordinal scale[19]

^*^also participated in member check focus group meeting.

#### RCT procedures measures

For quantitative measurements, descriptive analyses were performed. Descriptive data and proportions are presented for recruitment rates, retention rates, response rates, compliance to the allocated interventions, measurement completion, as well as the amount and nature of missing values. As this was a feasibility study with a rather small sample size, missing values were not imputed.

#### Quantitative outcome measures

Outcome data on accessibility and transfer of health information are presented for the total group and intervention- and control group separately. Variance within data is displayed by presenting the proportions per category for categorical variables and the mean and standard deviation (SD) for continuous variables, in relation to a total range of the scales.

#### Qualitative measures

Qualitative analysis was performed by a multidisciplinary research team (MW, FF, CB, JZ and NB), embodying expertise in both quantitative and qualitative research. The team included three medical doctors with experience in CHC practice, a health scientist and an epidemiologist.

Transcripts were analysed using the software program NVivo 12 Pro [28]. Two researchers (MW and JZ or NB or CB), independently explored and analysed the data retrieved from the interviews/focus groups, after which they discussed the findings to reach consensus. After each round of analysing 3 to 5 interviews, a discussion took place within the whole research team to reflect on the data and analyses, to broaden the analytical scope if it seemed necessary, and to decide on further sampling or adapting topic lists. The first author (MW) wrote reflective memos. The qualitative analysis comprised a constant comparative approach, which started with the open coding of relevant text fragments during an inductive phase. After analysing three interviews, axial coding was performed by arranging, renaming and/or relating codes to each other and identifying and pragmatically structuring categories. Then, during a more abductive phase, selective coding was conducted by the research team by relating the data to knowledge from the literature and to the quantitative findings. During this phase, overarching core concepts and themes were identified and codes and categories were restructured [29, 30]. After concluding that no new and relevant elements were generated anymore, the team decided to describe the findings and validate them during a “member check” focus group meeting [26, 27] with CHC professionals.

## Results

### Flow and baseline characteristics of participants

From the 192 eligible and invited CHC professionals, 39 CHC professionals volunteered to participate, of which 38 completed baseline measurements and started recruiting parents and adolescents and involved other caregivers.

**Fig. 1:**
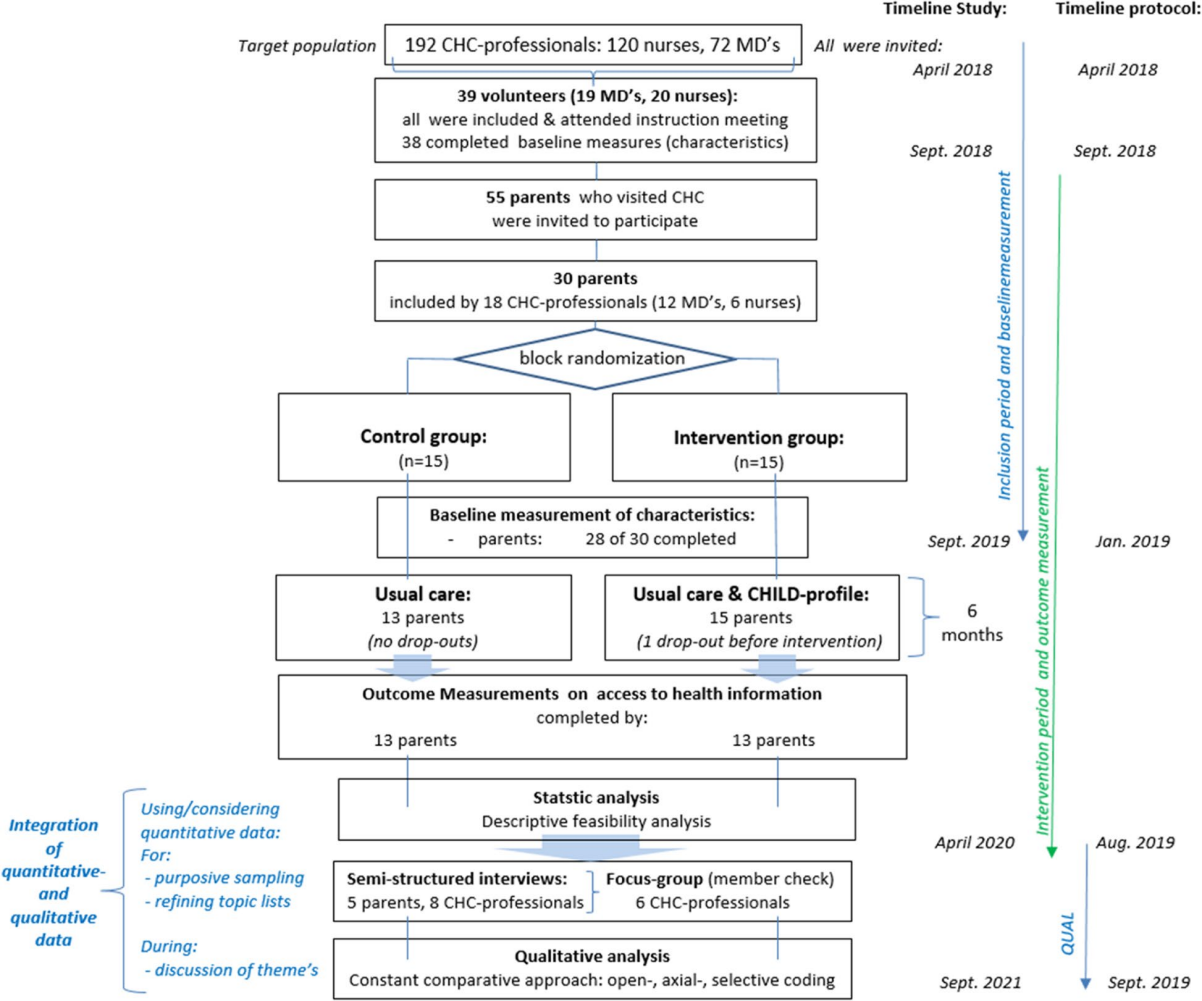
Participant flow throughout the study. (CHC = preventive Child Health Care, MD = medical doctor)

In total, 30 parents were included by 18 CHC professionals. The participant flow throughout the study period is presented in Fig. 1. As only one adolescent and one other caregiver were initially included, it was decided not to present their data.

The total group of included CHC professionals (*n*=38) and subgroup of professionals who included ≥ 1 parent (*n*=18) were both heterogeneous regarding discipline, target group (age children), education level and experience (see Table 1). On the forehand, most participating professionals were known with and positive about the 360°CHILD-profile but had no experience with using it in daily practice.

CHC professionals mostly used their computer and e-mail to provide information to parents and sometimes a tablet and/or WhatsApp for this purpose.

The subgroup of 18 CHC professionals, who included parent(s) and thus actively participated during the intervention period, consisted of twelve medical doctors and six nurses. The baseline characteristics of CHC professionals (of the total- and sub-group of professionals) are presented in Table 1.

Participating parents were mostly mothers (1 father) and Dutch (2 non-Dutch) and were rather positive to very positive about the provided care by CHC. The group of participating parents was heterogeneous regarding their education level, their children’s level of functioning and experienced problems, and their parental stress. The baseline characteristics of parents in the intervention group were rather comparable with those in the control group and total group (see Table 2).

Ten CHC professionals were invited for the semi-structured interviews. Eight participated and two professionals declined (one was too busy; one did not provide a reason). Five of the participating parents were invited and participated in an interview. Characteristics of the parents and CHC professionals are displayed in Table 3.

All interviewed CHC professionals were invited for a member check focus group meeting, of which six participated. Two professionals (Table 3) could not attend the meeting due to other obligations.

### RCT procedure measures

#### Recruitment

Recruitment rates are presented in Table 4. Indicated reasons for invited parents deciding not to participate were too busy (*n*=11), concerns about privacy due to online availability of health data (*n*=3), no expected added value of the 360°CHILD-profile (*n*=2), language barrier (*n*=2), already disappointed regarding offered CHC care (*n*=1), and non-acceptance of randomisation (*n*=1). For five invited parents, the reason not to participate could not be verified.

**Table 4:** Recruitment-, response- and retention-rates, measure completion

**Recruitment rates**	**Percentage** (number included participants/ number invited to participate)
CHC*-professionals	20% (39/192)
Parents	55% (30/55)
**Response rates**	**Percentage** (number returned/ number sent questionnaires)
Baseline measurements:	
- CHC*-professionals	97 % (38/39) (regarding their own characteristics)
	100% (30/30) (child’s level of functioning/experienced problems)
- Parents	96% (28/30)
Outcome measurements by parents	93% (26/28)
**Missing values**	**Percentage**
Baseline measurements:	
- CHC*-professionals	0.2% (regarding their own characteristics)
	5% (child’s level of functioning/experienced problems)
- parents	0.3%
Outcome measurements by parents	2%
**Retention rate**	**Percentage** (number of participants finishing RCT/number of included participants)
- CHC*-professionals	44% (17/39) (regarding total group)
	94% (17/18) (regarding subgroup of professionals who included parents)
- parents	87% (26/30)

^*^CHC: preventive Child Health Care

#### Response to measurements

For baseline measurements, response rates are presented in Table 4. The reason for parents’ non-response to outcome measurements was that they were too busy.

#### Measurement completion

The percentages of missing values in returned questionnaires were between 0.2 and 5% (Table 4). The missing values mostly concerned questions that were of low importance regarding the main topics of this study.

#### Retention

Retention rates are presented in Table 4. Lost to follow-up of parents enclosed not responding to baseline questionnaire (*n*=2) or outcome questionnaire (*n*=1) and not responding to the invitation for the CHC consultation in which the intervention would be performed (*n*=1). Within the group of CHC professionals, one did not return the questionnaire at baseline, and 20 could not start or finish the intervention period because they did not succeed in including a parent and/or quit working at the CHC. Of the CHC professionals who did include a parent and started the intervention period, 94% finished their tasks within RCT.

#### Compliance to the allocated intervention

There was one case of non-compliance to intervention in the experimental group. The mother cancelled her CHC appointment after randomisation.

#### Adaptation of RCT procedures

The inclusion of parents remained behind schedule. After prolonging the original recruitment period (of four months) by 2 months, 15 CHC professionals were active in recruiting parents and 16 parents signed informed consent. Therefore, the recruitment period was prolonged by another 6 months and three additional activities were executed to stimulate recruitment: (1) a poster with information about the study was distributed, (2) four students from the Maastricht Medical School were trained to support CHC professionals during recruitment by observing consultation hours and providing eligible parents with information on the study right after the visit, and (3) CHC professionals were provided with extra information, reminders, and advice (via e-mail) on how to enhance recruitment.

The 360°CHILD-profile appeared to be not yet fully integrated within the EMD and online CHC portal for parents. Therefore, the personal 360°CHILD-profiles had to be completed by hand by the researcher and an internal part of the CHC website (secured with sms authentication) had to be used to provide parents with online access to their child’s 360°CHILD-profile.

### Quantitative outcome measures

Outcomes of the used outcome measures (Consumer Quality Index (CQI), supplemental items on Health Information Technology (HIT) and additional original questions on accessibility of health care and information) showed that for the majority of the items, 75–100% of the parents chose positive answer options, while negative answer options were chosen by 0–25% of the parents (see Tables 5, 6, and 7). Items 6–15 out of the HIT were appointed as not applicable by the majority of parents (Table 6).

**Table 5:** Outcome of consumer quality index for parents

**Items Consumer Quality Index for parents**	**Answer options**	**Total group *N*=28 Number (%)**	**Intervention group (CHILD-profile) *n*=15 Number (%)**	**Control group *n*=13 Number (%)**
1. Before the CHC-visit, was it clear for you what to expect from the consultation?	No	4 (15)	3 (23)	1 ( 8)
	Yes	22 (85)	10 (77)	12 (92)
	Missing values	2	2	
2. Did you receive advice during the visit?	No	6 (23)	4 (31)	2 (15)
	Yes	20 (77)	9 (69)	11 (85)
	Missing values	2	2	0
3. Was the advice from CHC applicable in your family situation?	No	0	0	0
	Yes	20 (100)	9 (100)	11 (100)
	Not applicable	8	6	2
4. In what extend was it a problem to reach contact with CHC by telephone?	A big problem	1 ( 6)	1 (13)	0
	A small	4 (23)	3 (37)	1 (11)
	No problem	12 (71)	4 (50)	8 (89)
	Not applicable	9	5	4
	Missing values	2	2	0
5. In the past 6 months, did you send CHC an e-mail to ask CHC a question?	No	22 (85)	13 (100)	9 (69)
	Yes	4 (15)	0	4 (31)
	Missing values	2	2	0
6. Did you timely receive a reply on your mail?	Never	0		0
	Sometimes	1 (25)		1 (25)
	Often	0		0
	Always	3 (75)		3 (75)
	Not applicable	24	15	9
7. Did you receive an answer to your question?	Never	0		0
	Sometimes	1 (25)		1 (25)
	Often	0		0
	Always	3 (75)		3 (75)
	Not applicable	24	15	9
8. Did you have contact with a nurse and/or a MD?	No	13 (48)	5 (38)	8 (67)
	Yes	12 (52)	8 (62)	4 (33)
	Missing values	3	2	1
9. Was the MD/nurse kind?	No	0	0	0
	Yes	12 (100)	8 (100)	4 (100)
10. Was the MD/nurse competent?	No	0	0	0
	Yes	12 (100)	8 (100)	4 (100)
11. Did you experience room to ask questions?	No	0	0	0
	Yes	12 (100)	8 (100)	4 (100)
12. Did you experience room to share your view?	No	0	0	0
	Yes	12 (100)	8 (100)	4 (100)
13. Did the MD/nurse provide good answers to your questions?	No	0	0	0
	Yes	12 (100)	8 (100)	4 (100)
14. Did the MD/nurse provide comprehensible explanation?	No	0	0	0
	Yes	12 (100)	8 (100)	4 (100)
15. Was the MD/nurse well informed about the medical history of the child?	No	1 (10)	0	1 (25)
	Yes	9 (90)	6 (100)	3 (75)
16. Were you referred well to other caregivers, if needed?	No	1 ( 7)	1 (13)	0
	Yes	14 (93)	7 (87)	7 (100)
	Not applicable	13	7	6
17. Did you have contact with other caregivers with regard to your child?	No	12 (48)	7 (58)	5 (39)
	Yes	13 (52)	5 (42)	8 (61)
	Missing values	3	3	
18. Did CHC collaborate well with other caregivers?	No	3 (12)	2 (17)	1 ( 8)
	Yes	22 (88)	10 (83)	12 (92)
	Missing values	3		
19. Are you sufficiently informed about the extra service the CHC offers?	No	11 (46)	6 (55)	5 (38)
	Yes	13 (54)	5 (45)	8 (62)
	Missing values	4	4	0
20. Would you desire a broader service to be offered by CHC?	Yes	0	0	0
	No	24 (100)	11 (100)	13 (100)
	Missing values	4		
21. Was it clear for you what you could expect from CHC?	No	4 (15)	3 (23)	1 ( 8)
	Yes	22 (85)	10 (77)	12 (92)
	Missing values	2	2	0
22. Would you recommend CHC for other parents?	Definitely not	0	0	0
	Probably not	2 ( 8)	1 ( 8)	1 ( 8)
	Probably yes	16 (64)	7 (59)	9 (69)
	Definitely yes	7 (28)	4 (33)	3 (23)
	Missing values	3	3	0
23. Overall score for CHC		(mean, SD)	(mean, SD)	(mean, SD)
	Score 0-10	7.68 (1.15)	7.83 (0.94)	7.54 (1.33)

**Table 6:** Outcome of the supplemental health information technology-items from the CAPHS ©-website

**Supplemental items from Health Information Technology**	**Answer options**	**Total group Number (%)**	**CHILD-profile group Number (%)**	**Control group Number (%)**
1. Did medical doctor/nurse use a computer, smartphone or tablet used during visit?	No	10 (38)	7 (54)	3 (23)
	Yes	16 (62)	6 (46)	10 (77)
	Missing values	2	2	0
2. Has medical doctor/nurse looked up information?	No	4 (27)	0	4 (40)
	Don’t know	5 (33)	2 (40)	3 (30)
	Yes	6 (40)	3 (60)	3 (30)
	Not applicable	13	10	3
3. Did medical doctor/nurse show information?	No	3 (19)	0	3 (30)
	Yes	13 (81)	6 (100)	7 (70)
	Not applicable	12	9	3
4. Was the use of the computer useful?	No	0	0	0
	Yes, a little bit	3 (19)	0	3 (30)
	Yes, absolutely	13 (81)	6 (100)	7 (70)
	Not applicable	12	9	3
5.The use of the computer made communication:	Harder	0	0	0
	Not harder/not easier	7 (44)	1 (17)	6 (60)
	Easier	9 (56)	5 (83)	4 (40)
	Not applicable	12	9	3
6. Did CHC make information online accessible?	No:	9 (35)	3 (23)	6 (46)
	Don’t know:	11 (42)	4 (31)	7 (54)
	Yes	6 (23)	6 (46)	0
	Missing values	2	2	0
7. Did you look up information during the last 6 months?	No	5 (71)	5 (71)	0
	Yes	2 (29)	2 (29)	0
	Not applicable	21	8	13
8. If so, how often did you look up information?	1-2x	1 (50)	1 (50)	0
	3-4x	1 (50)	1 (50)	0
	5-6x	0	0	0
	>6x	0	0	0
	Not applicable	26	13	13
9. How easy was it to find information?	Very easy	1 (50)	1 (50)	0
	Rather easy	1 (50)	1 (50)	0
	Not very easy	0	0	0
	Not easy at all	0	0	0
	Not applicable	26	13	13
10. How understandable was the given information?	Very	0	0	0
	Rather	2 (100)	2 (100)	0
	Not very	0	0	0
	Not at all	0	0	0
	Not applicable	26	13	13
11. To whom did you show the information?	Nobody	2 (100)	2 (100)	0
	Family member(s)	0	0	0
	Caregiver(s)	0	0	0
	Other(s)	0	0	0
	Not applicable	26	13	13
12. Did CHC give you access to conclusions?	No:	15 (56)	5 (38)	10 (77)
	Don’t know:	10 (40)	7 (54)	3 (23)
	Yes:	1 ( 4)	1 ( 8)	0
	Missing values	2	2	0
13. How did CHC give you access to conclusions?	On paper	1 (100)	1 (100)	0
	Via internet	0	0	0
	Via email	0	0	0
	Other way	0	0	0
	Not applicable	27	14	13
14. Did you read the conclusions?	No	0	0	0
	Yes	1 (100)	1 (100)	0
	Not applicable	27	14	13
15. How understandable were the conclusions?	Very	1 (100)	1 (100)	0
	Rather	0	0	0
	Not very	0	0	0
	Not at all	0	0	0
	Not applicable	27	14	13

**Table 7:** Outcome of added original questions on accessibility of health-care and information

**Additional questions**	**Answer options**	**Total group (*n*=28) Number ( %)**	**CHILD-profile group Number (%)**	**Control group Number (%)**
1. I know for what questions/problems I can contact the CHC	”I totally disagree”	0	0	0
	“I disagree”	3 (12)	2 (15)	1 ( 8)
	“I do not agree/not disagree”	0	0	0
	“I agree”	17 (65)	8 (62)	9 (69)
	“I totally agree”	6 (23)	3 (23)	3 (23)
	Missing values	2	2	
2. It is clear for me how to contact CHC for questions etc.	”I totally disagree”	0	0	0
	“I disagree”	1 ( 4)	0	1 ( 8)
	“I do not agree/not disagree”	2 ( 7)	1 ( 8)	1 ( 8)
	“I agree”	14 (54)	8 (61)	6 (46)
	“I totally agree”	9 (35)	4 (31)	5 (38)
	Missing values	2	2	
3. The way I get advice/information from CHC fits my needs	”I totally disagree”	0	0	0
	“I disagree”	1 ( 4)	0	1 ( 8)
	“I do not agree/not disagree”	4 (15)	0	4 (31)
	“I agree”	18 (69)	11 (85)	7 (53)
	“I totally agree”	3 (12)	2 (15)	1 ( 8)
	Missing values	2	2	
4. If I have questions, it is easy for me to get in contact with CHC.	”I totally disagree”	0	0	0
	“I disagree”	1 ( 4)	1 ( 8)	0
	“I do not agree/not disagree”	4 (15)	2 (15)	2 (15)
	“I agree”	16 (62)	7 (54)	9 (70)
	“I totally agree”	5 (19)	3 (23)	2 (15)
	Missing values	2	2	
5. The CHC radiates trust and a positive ambiance.	”I totally disagree”	0	0	0
	“I disagree”	1 ( 4)	0	1 ( 8)
	“I do not agree/not disagree”	6 (23)	4 (31)	2 (15)
	“I agree”	13 (50)	6 (46)	7 (54)
	“I totally agree”	6 (23)	3 (23)	3 (23)
	Missing values	2	2	
6. I am a person who, when having concerns and/or questions, quickly asks for advice and/or help.	”I totally disagree”	0	0	0
	“I disagree”	6 (23)	3 (23)	3 (23)
	“I do not agree/not disagree”	7 (27)	2 (15)	5 (38)
	“I agree”	10 (38)	6 (47)	4 (31)
	“I totally agree”	3 (12)	2 (15)	1 ( 8)
	Missing values	2	2	

### Qualitative measures

During qualitative analysis, while reflecting on both qualitative and quantitative data to gain a deeper understanding of the quantitative findings regarding the feasibility of performing an RCT, five categories emerged: “Interest, willingness and self-efficacy regarding study participation”, “Emerging difficulties with the recruitment of parents by CHC professionals”, “Overall study participation, randomisation and intervention”, “Points for improvement of RCT-procedures” and “Outcome measures on accessibility and transfer of health information”.

For each category, findings are described and related quotes are presented in a box.

#### Interest, willingness and self-efficacy regarding study participation

Quantitative findings showed that within the given period, a sufficient number of CHC professionals volunteered to participate in the study. During the interviews, CHC professionals and parents said they were interested in the 360°CHILD-profile and willing to help. Professionals were satisfied with the clearness of the provided instructions and, on the forehand, felt capable to recruit enough parents and perform their study tasks.

**Table:** 

**Box 1: Quotes related to “Interest, willingness and self-efficacy regarding study participation”.** Parent 2: *“I was very interested and it was really nice to see the mapped health information”.* CHC-professional 4: *“I see added value and my colleagues also have a warm heart for the 360°CHILD-profile”.* CHC-professional 1: *“After the instruction meeting, I thought it would be very easy because every professional only had to find a few parents”.* *CHC-professional 8:* *“My thought was that it should work, I will at least do that. That thought came from a feeling of commitment, dedication and seeing the value of the 360°CHILD-profile.”*	

#### Emerging difficulties with the recruitment of parents by CHC professionals

During the RCT, recruitment of parents by CHC professionals appeared to be seriously hindered. Most CHC professionals mentioned a high workload due to a lack of staff and time. They prioritised tasks directly related to the regular care of children. Some professionals had a clear picture about the specific target group to recruit while others felt a bit uncertain about that. Some CHC professionals mentioned that the 360°CHILD-profile was new for them, as it was not yet fully integrated within the EMD. This made it harder to inform parents about the 360°CHILD-profile and made parents reluctant to participate.

A small number of professionals mentioned they felt some reluctance to burden parents who already experience substantial problems concerning their child’s upbringing. A few CHC professionals mentioned they tended to ask parents they were on good terms with.

**Table:** 

**Box 2: Quotes related to: “Emerging difficulties with recruitment of parents by CHC-professionals”** CHC-professional 3: *“It faded away from my attention and due to low staff capacity, I already had to do extra work and couldn’t find time to fill in a questionnaire. That was frustrating.”* *CHC-professional 7:* *“My job is very busy and at the end of the day I have to prioritise. Then I mostly choose finishing urgent tasks related to clients.”* *“The 360°CHILD-profile is still new and unknown for parents, which made them reluctant. Once it would be fully integrated within CHC, I’m sure parents would like it.”* CHC-professional 2: *”During implementation, we as professionals should be provided with extra time, but after a while it will make us finish work faster”.* CHC-professional 1: *“I did not want to ask parents, who experienced severe problems because I wondered if they would have time for it and if the burden would be in balance with the added value for them.”*	

#### Overall study participation, randomisation and intervention

Qualitative data showed congruence with the positive quantitative findings on retention, randomisation and intervention. Parents and CHC professionals mentioned the study procedures were clear and easy. They were positive about the provided communication and reminders by researchers. Parents who participated in the study said that the randomisation process was clear and acceptable. However, one of the recruited parents decided (before randomisation) not to participate because she would not know if she would receive the 360°CHILD-profile during the study period. Parents appreciated the intervention, and once they found time for their study tasks, they did not perceive these tasks as a major burden.

**Table:** 

**Box 3: Quotes related to “Overall study participation, randomisation and intervention”.** Parent 3: *“The study participation did not burden me. It was actually very nice that my child’s health information is presented on a profile.”* *“It was clear for me that I would receive the 360°CHILD-profile immediately of after 6 months. I would not know why that would be a problem.”* Parent 1: *“It all was clear and went well. No difficulties.”* CHC-professional 5: *“The instructions were very clear and it was nice to receive the instruction map. Very professional”.* CHC-professional 8: *“Presenting the 360°CHILD-profile went well. It fits my way of working and it was clear for parents.”* CHC-professional 6: *“My participation didn’t cost me much extra time. It actually went very well”.*	

#### Points for improvement of RCT procedures

The support of trained students, which was initiated when recruitment appeared to be difficult, was very much appreciated and improved recruitment. Qualitative data also revealed additional considerations for improving RCT procedures (like using social media and invitation letters for regular CHC-visits, rehearsing the presentation of the 360°CHILD-profile shortly with researcher and/or colleagues, and sufficiently facilitating professionals to familiarize with the intervention).

**Table:** 

**Box 4: Quotes related to “Points for improvement of RCT-procedures”.** Parent 5: *“You could also use social media or newsletters from schools to recruit parents.”* CHC-professional 5: *“Maybe inform all parents by a letter, prior to the CHC-visit. Then, during the visit I can ask if they did read the letter.”* CHC-professional 3: *“The student’s support was great, a big relief. When she informed parents, I could do other tasks”.* CHC-professional 4: *“Well, presenting the 360°CHILD-profile for the first time felt challenging. It would have been a good idea if I had taken the researcher up on her offer to firstly discuss it together.”* CHC-professional 6: *“If we would have rehearsed with the 360°CHILD-profile within small groups of colleagues, that would have yielded more binding with the innovation and motivation to use it”.* CHC-professional 2: *“Take time for implementation, so professionals can familiarise with the 360°CHILD-profile.”* CHC-professional 1: *“Keep evaluating the 360°CHILD-profile during implementation.”*	

#### Outcome measures on accessibility and transfer of health informations

Interviewed parents mentioned that completing the questionnaire was only a little effort. They found the questionnaire acceptable and comprehensible. A few parents explained why they chose certain answer options. However, not all parents could explicitly remember which questionnaire it concerned. During the study period, they also received other questionnaires related to usual care and related to a new digital parent portal.

**Table:** 

**Box 5: Quotes related to “Outcome measures on accessibility and transfer of health information”.** Parent 4: *“The questionnaire was all right: nothing difficult or taxing and I finished it pretty quickly.”* Parent 2: *“I completed several questionnaires for the CHC. I can’t remember which one came from you.”* *“On the question if CHC made health information available via a website I chose answer option “no”. But, yes indeed, the 360°CHILD-profile was online available. I got an e-mail with a link and code and got secured access to an online portal. I would call that an online environment and not via a website.”* Parent 3: *“Yes, I chose answer option “no” for the question if I have had contact with the CHC-nurse or medical doctor. I thought that that only counted for extra contacts when something was wrong, not a regular CHC-contact.”*	

## Discussion

This feasibility RCT was a first step towards evaluating 360°CHILD-profile’s effectiveness. It provided insight in the complexity of performing an RCT within the organizational CHC context. The use of a mixed methods approach enabled to thoroughly investigate feasibility of RCT procedures (objective 1) and the applicability of potential outcome measures for studying 360°CHILD-profile’s impact on access and transfer of health information (objective 2).

With regard to the first objective, positive findings were generated on practical feasibility of the used randomisation schedule, measurements and experimental intervention (the 360°CHILD-profile) within the CHC. However, recruitment of parents by CHC professionals appeared to be problematic and was hindered by organisational factors within the CHC-context.

Regarding the second objective, the used outcome measures showed skewed results consisting of high percentages of positive scores in both groups. In addition, outcomes revealed low applicability of, by the researchers beforehand assumed as relevant, items on accessibility and transfer of health information (Table 6, HIT items 6–15).

Overall, integrative findings revealed that conducting a robust RCT design within the given context will probably be even more complex, time-consuming and costly than initially expected.

This research project identified several hindering organizational factors like the organizational structure (within each geographically sub-region, one medical doctor and one nurse are responsible to provide care to all the children living in that sub-region), which influences the applicability of the preferred randomisation method. Next, the research project (which concerned both 360°CHILD-profile’s implementation and evaluation) was insufficiently prioritised and facilitated by CHC management, which hindered recruitment of parents by CHC professionals.

### Randomisation

The inevitable choice of randomisation on the level of individual parents led to the possibility that CHC professionals who included more parents were to perform both the usual care and the experimental intervention. Although within this feasibility study, participating professionals did not perceive this as problematic, this situation should be avoided in a future RCT as much as possible. Cluster randomisation might be required and consequently, more complex analysis and much larger sample sizes.

### Emerging difficulties with recruitment of parents by CHC professionals

The CHC professionals seemed motivated and felt capable to recruit parents. Although motivation and self-efficacy are facilitators for recruitment [31–33], in daily practice, recruitment appeared to be more problematic then expected by the CHC professionals. It could be that, on forehand, professionals overestimated their motivation and self-efficacy and tended towards socially desirable answers. However, study findings also revealed hindering contextual factors like insufficient prioritisation and facilitation by CHC management. This led to a perceived high workload and prioritisation of daily care tasks by CHC professionals. They tended to postpone tasks related to participating in this study, which they seemed to perceive as tasks, less fitting to their job profile. CHC professionals were not yet familiar with this intervention due to the lack of a technical integration of the 360°CHILD-profile within the EMD.

It was known that the used recruitment strategy (CHC professionals recruiting parents) was not the optimal option [34]. However, researchers anticipated that recruiting the restricted number of one to two parents by each professional should be doable. This was enforced by the, on forehand, enthusiastic reactions and positive expectations of participating professionals. Study participation was expected not to be perceived as burdensome because the intervention was assumed to fit the CHC working method based on former results [1, 6]. Nevertheless, some professionals appeared to expect the whole research process to be a possible burden to parents who experienced problems around their child’s upbringing. They seemed reluctant to ask parents in problematic situations to participate in the study. Next, qualitative data led to the impression that CHC professionals preferred inviting parents they were in good terms with. These findings provided insight in how professionals’ relationship with parents, as their caregivers, influenced recruitment, potentially leading to selection bias.

This study enabled to adopt an alternative and more successful strategy for recruiting parents; deploying trained research staff for this task. Research staff, independent from CHC-care, is likely more equipped to support parents during their decision-making process whether to participate and sign informed consent [31, 34]. However, it must be taken into account that for large studies, this strategy requires substantial more research staff and a complex planning.

### Outcome measures

Outcome measures revealed severe skewness to one direction (positive answer options) which might limit the interpretation of data. Moreover, the number of relevant items on accessibility and transfer of health information appeared to be rather limited and oftentimes not applicable for a substantial part of the participants. The used outcome measures were validated within the CHC to measure accessibility of the CHC [11, 22]. This theoretical construct might have been formulated with too less detail to be sufficiently applicable for evaluating 360°CHILD-profile’s impact on the access and transfer of health information within CHC.

### Strengths and limitations

Integration of quantitative and qualitative data enabled to strengthen the validity of findings through triangulation [35]. More approaches were incorporated to strengthen trustworthiness of the qualitative findings [35]. To enhance researchers’ reflexivity during qualitative analysis, the original research team (MW, CB, FF) was expanded by two researchers, external of the project so far (JZ, NB). They often played a role as critical reviewer and questioned methods and researchers’ interpretations and assumptions. Researchers repeatedly returned to the raw data and memos to search for consistent and/or disconfirming data regarding interpretations and categories. Finally, participants were given the opportunity to correct and react on researchers’ interpretations (member check focus group).

The rather heterogeneous group of parents and CHC professionals and purposive sampling for interviews enabled to consider a variety of perspectives. Insight in these perspectives led to deeper understanding of quantitative data and identification of hindering contextual factors within the CHC organisation and uncertainties concerning applicability of outcome measures. Moreover, valuable qualitative data led to better insight in how organisational factors influenced RCT procedures and how to improve these procedures. The engagement of stakeholders, consideration of context, identification of uncertainties and refinement of theory are elements that are identified as core elements for evaluating complex interventions [36]. These core elements are described in a recent publication of Skivington, who presents a new framework for development and evaluation of complex interventions [36].

Although valuable qualitative data were generated within this study, this was rather limited with regard to gaining deeper understanding on the outcomes of the used outcome measures. Probably this was influenced by the time passed between measure completion and the interviews and parents’ confusion with other questionnaires that were send out during the same period. During qualitative analysis, it became clear that the time-span between the active participation of the parents and the interview more often led to rather a limited extend of memories of parents about their study participation and questionnaires. Therefore, it was decided that performing a member check focus group with parents would not yield substantial new, more in-depth insights regarding this phase of the evaluation process.

Other limitations were the rather small study population and the fact that participants might have been relatively more positive about CHC and the 360°CHILD-profile.

### Future research

This study revealed the importance of considering the specific CHC context when designing future research. This organisational context requires a more complex randomisation strategy and, consequently, larger sample sizes. Next, an active role of management should not be underestimated in order to facilitate CHC professionals sufficiently. Preferably, professionals should be provided with sufficient time, recurrent communication, pro-active and continuous support, training and opportunities to rehearse study tasks with colleagues [32, 33]. Thereby, management should properly prioritise the ICT integration within the currently used EMD, which helps professionals to familiarise with the 360°CHILD-profile. Foremost, it is essential that professionals are capable to perform the intervention, while recruitment of parents can and preferably should be performed by trained research staff, independent from CHC care.

Finally, further evaluation of 360°CHILD-profile’s implementation and future effectiveness within CHC requires a thorough search for and/or development of appropriate outcome measures. Potential measures must be extensively investigated by using qualitative and subsequently quantitative clinimetric methods.

During next implementation phases, the focus will be firstly on identifying outcome measures for valid assessment of 360°CHILD-profile’s impact on the accessibility and transfer of health information. After full implementation, additional outcome measures should be identified: outcome measures that will be applicable for evaluating expected potential impact of the 360°CHILD-profile on the quality of shared decision-making and most importantly the complex preventive clinical reasoning within the CHC [1]. Namely, the 360°CHILD-profile is designed to enable parents to be more actively involved in decision-making processes and to intuitively guide thinking processes of all stakeholders in line with the biopsychosocial concept of health and personalised health care [1, 37].

Based on the integrative findings of this pragmatic feasibility RCT, it should be questioned whether an RCT is the most appropriate design for the future steps of the 360°CHILD-profile’s implementation and evaluation process within CHC practice. The research group of Skivington supports the questioning of an RCT design within the setting of evaluating complex interventions [36]. The strive for executing an RCT for gaining evidence on effectiveness should not lead to postponing and/or hindering implementation of promising interventions. In case of the 360°CHILD-profile, consistent positive findings on usability and benefits for CHC practice so far, justify a next step in the implementation process [36]. For complex interventions and settings, like the 360°CHILD-profile and the CHC setting, it might be equally, or even more essential to build a theory on how the intervention impacts practice and how the complex context influences outcomes [36]. This asks for a deliberate, flexible approach and consideration of alternative designs. A quasi-experimental design and mixed methods process evaluations must be considered with a focus on generating outcomes on implementation and/or impact in practice [36]. Gaining input from all stakeholders is important to enable identification of key uncertainties, mechanisms of change, important contextual factors and relevant outcome measures. Foremost, for complex interventions, knowledge should be generated that is needed for taking decisions on if and how to proceed the implementation and evaluation process [36].

## Conclusions

This mixed methods feasibility RCT was an essential and robust step within the iterative impact oriented downstream validation process of the 360°CHILD-profile. The study revealed how organisational factors within the CHC context interfere with the execution of an RCT with the aim of generating valid outcomes regarding intended goals. This context would require a more complex randomisation strategy and the deployment of trained research staff for recruiting parents. Measures, potentially for evaluating 360°CHILD-profile’s implementation and effectiveness must be further explored and thoroughly piloted before proceeding the evaluation process.

Overall, integrative findings led to questioning the RCT as the most appropriate design for evaluating 360°CHILD-profile’s effectiveness within the CHC-context. Preparing for and executing an RCT is expected to be very complex and time-consuming and could hinder implementation of this promising innovation with obvious benefits for CHC practice. Alternative designs and mixed methods research must be considered during next implementation phases. The focus should be on generating valuable knowledge for deciding if and how to proceed to the next phase within the implementation and evaluation process.

## Supplementary Information

**Additional file 1.**

## Data Availability

The datasets used and/or analysed during the current study are available from the corresponding author [MW] on reasonable request.

## Abbreviations

CAHPS: Consumer Assessment of Healthcare Providers and Systems
CGAS: Children’s Global Assessment Scale
CHC: Preventive child health care
CQI: Consumer Quality Index
EMD: Electronic medical dossier
HIT: Supplemental items on Health Information Technology
ICF-CY: International Classification of Functioning, Disability and Health for Children and Youth
NOSIK: Dutch short version of parenting Stress Index
RCT: Randomised controlled trial
STEP: Dutch standardised professional's rating of child's functioning
